# Crystal structure of [μ_2_-3,3-dimethyl-4-(propan-2-yl­idene)thietane-2,2-dithiol­ato-κ^4^ *S*:*S*′:*S*:*S*′]bis[tricarbonyl­iron(I)](*Fe*—*Fe*)

**DOI:** 10.1107/S2056989015018496

**Published:** 2015-10-10

**Authors:** Peihua Zhao, Jeffery A. Bertke, Thomas B. Rauchfuss

**Affiliations:** a School of Chemical Sciences, University of Illinois at Urbana-Champaign, Urbana, Illinois 61801, USA

**Keywords:** crystal structure, iron(I), thietane, hexa­carbon­yl

## Abstract

The title complex, [Fe_2_(C_8_H_12_S_3_)(CO)_6_] or [{Fe(CO)_3_}_2_(μ-*L*)] [*L* = 3,3-dimethyl-4-(propan-2-yl­idene)thietane-2,2-bis­(thiol­ato)], consists of two Fe(CO)_3_ moieties double-bridged by a di­thiol­ate ligand. Each of the two Fe^I^ atoms has a distorted anti-prismatic coordination environment consisting of three carbonyl groups, two S atoms of the di­thiol­ate ligand and the neighboring Fe^I^ atom [Fe—Fe = 2.4921 (4) Å]. Weak C—H⋯O inter­molecular inter­actions result in the formation of dimers. This is the second crystal structure reported with the 3,3-dimethyl-4-(propan-2-yl­idene)thietane-2,2-bis­(thiol­ate) ligand and the first in which it bridges two metal atoms.

## Abstract 2

The dinuclear complex [{Fe(CO)_3_}_2_(μ-*L*)] [*L* = 3,3-dimethyl-4-(propan-2-yl­idene)thietane-2,2-bis­(thiol­ate)] consists of two Fe(CO)_3_ moieties bridged by a di­thiol­ate ligand. This is the first crystal structure reported in which the 3,3-dimethyl-4-(propan-2-yl­idene)thietane-2,2-bis­(thiol­ate) ligand bridges two metal atoms.

## Chemical context   

Iron–sulfur complexes have attracted considerable attention over the past decades (Ogino *et al.*, 1998 ▸). This is mainly because such complexes possess the distinctive iron–sulfur cluster core, which is biologically related to the active site of [FeFe]-hydrogenases (Fontecilla-Camps *et al.*, 2007 ▸). In particular, [FeFe]-hydrogenases are a class of natural enzymes that can reversibly catalyse the evolution and uptake of hydrogen in several microorganisms (Cammack, 1999 ▸; Stephenson & Stickland, 1931 ▸). In view of this, a large number of iron–sulfur cluster complexes have been designed and synthesized as the active site models of [FeFe]-hydrogenases (*e.g.* Capon *et al.*, 2005 ▸; Darensbourg *et al.*, 2000 ▸; Gloaguen & Rauchfuss, 2009 ▸; Rauchfuss, 2015 ▸; Tard & Pickett, 2009 ▸).

Most recently, we investigated the preparation of iron–sulfur complexes *via* the reaction of 1,3-cyclo­butane­dithiol­ate compounds with [Fe_3_(CO)_12_] and have obtained an unexpected iron–sulfur complex, [Fe_2_(CO)_6_(C_8_H_12_S_3_)] or [{Fe(CO)_3_}_2_(μ-*L*)] [*L* = 3,3-dimethyl-4-(propan-2-yl­idene)thietane-2,2-bis­(thiol­ate), C_8_H_12_S_3_], (I). 
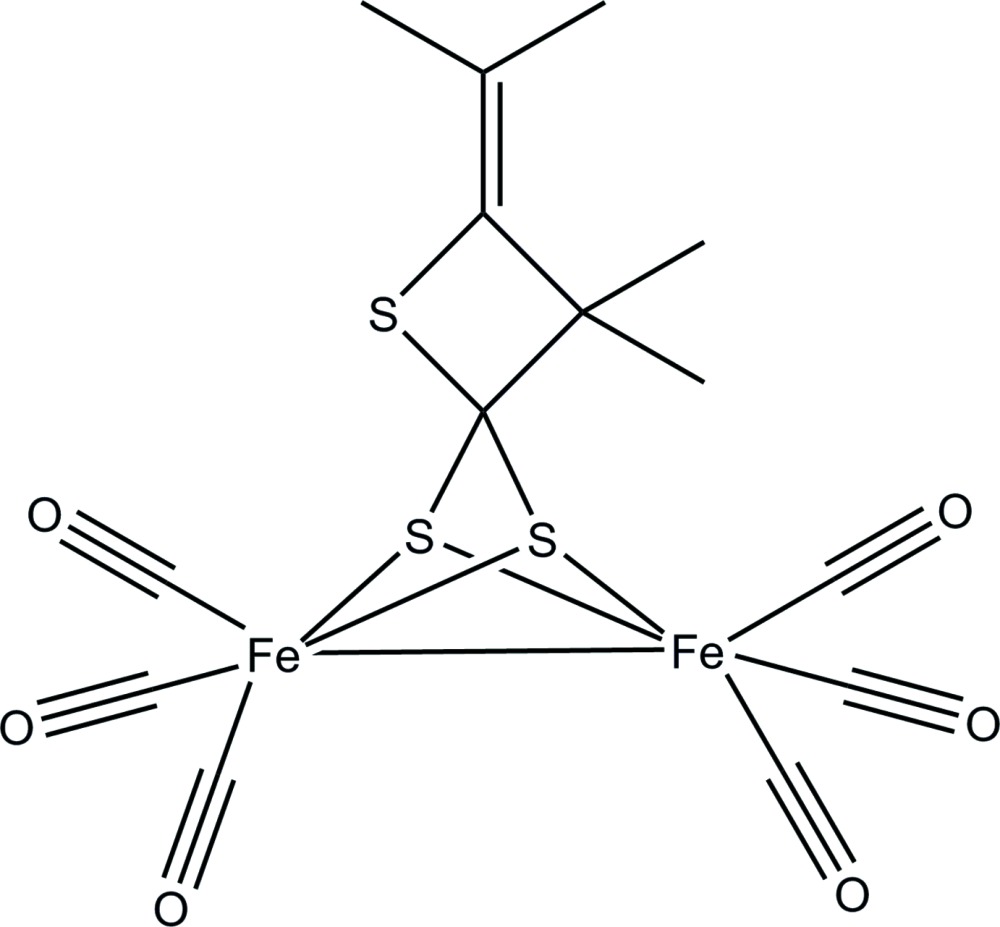


**Figure 1:**
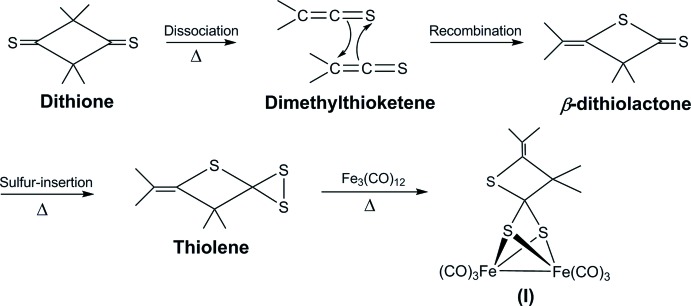
Schematic representation of a possible formation process for the 3,3-dimethyl-4-(propan-2-yl­idene)thietane-2,2-bis­(thiol­ato) ligand from the starting material.

Fig. 1 ▸ shows a possible formation process for the 3,3-dimethyl-4-(propan-2-yl­idene)thietane-2,2-bis­(thiol­ate) ligand *via* rearrangement of the di­thione starting material and its reaction to form compound (I). Similar rearrangements of di­thio­nes have been reported previously (Elam & Davis, 1967 ▸). Herein, we report the synthesis conditions and crystal structure of the title complex (I).

## Structural commentary   

**Figure 2:**
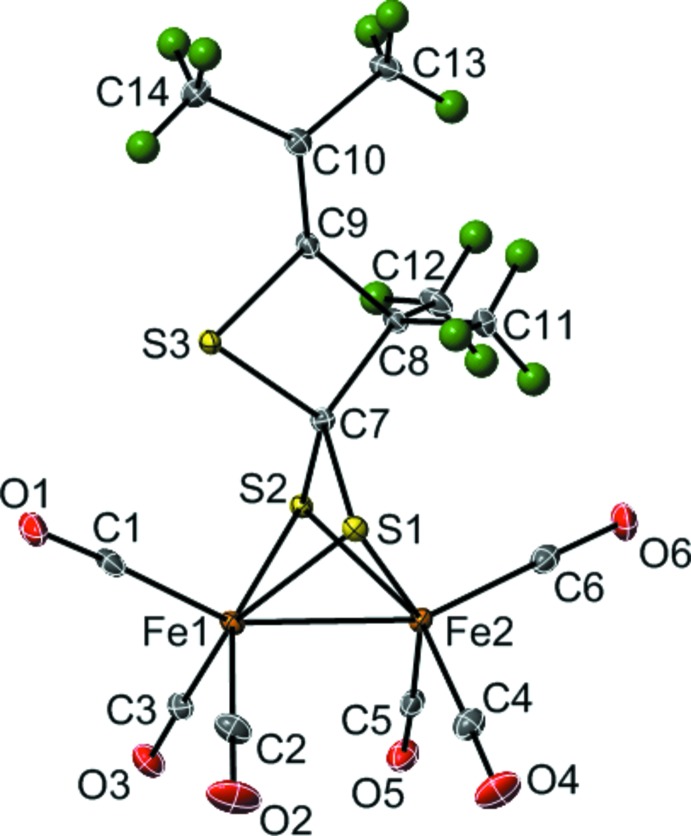
The mol­ecular structure of (I) with displacement ellipsoids drawn at the 35% probability level for non-H atoms and spheres of arbitrary size for H atoms.

The mol­ecular structure of (I) consists of two six-coordinate iron(I) atoms, each in a distorted trigonal anti-prismatic coordination environment (Fig. 2 ▸). The coordination sphere of Fe1 is filled by three carbonyl C atoms [Fe1—C1 = 1.8158 (19), Fe1—C2 = 1.7900 (18), Fe1—C3 = 1.8047 (18) Å), two S atoms of a bridging di­thiol­ate ligand [Fe1—S1 = 2.2675 (5), Fe1—S2 = 2.2636 (5) Å], and the neighboring Fe^I^ atom [Fe1—Fe2 = 2.4921 (4) Å]. The coordination sphere of Fe2 is similarly filled by three carbonyl C atoms [Fe2—C4 = 1.7986 (19), Fe2—C5 = 1.8013 (19), Fe2—C6 = 1.8054 (19) Å], two S atoms [Fe2—S1 = 2.2624 (5), Fe2—S2 = 2.2601 (5) Å], and the neighboring Fe^I^ atom.

The C7—S3—C9 bond angle of 77.86 (8)° is significantly smaller than the other angles making up the thietane ring [S3—C7—C8 = 92.82 (10)°; S3—C9—C8 = 96.26 (11)°; C7—C8—C9 = 93.06 (12)°]. The central ring of the anion is nearly planar with a S3—C7—C8—C9 torsion angle of −0.74 (11)°. The plane through S1—C7—S2 is rotated by 89.94 (11)° with respect to the thietane ring. Similarly, the dihedral angle between the thietane ring and the plane through C11—C8—C12 is 89.74 (16)°. The =C(CH_3_)_2_ group (C13—C10—C14) is only slightly out of the plane of the central ring, making a dihedral angle of 4.63 (18)°.

## Supra­molecular features   

**Figure 3:**
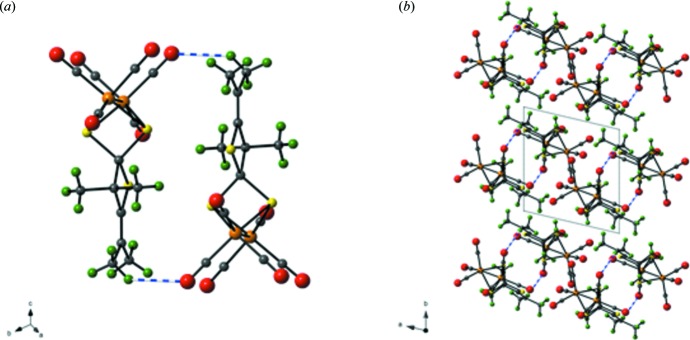
A plot of (*a*) dimers of (I) with the C—H⋯O inter­actions highlighted as blue dashed lines; and (*b*) an expanded view along the *c* axis of the packing of (I) with an overlay of the unit cell. Orange = Fe, yellow = S, red = O, gray = C, green = H.

There are no significant supra­molecular features to discuss with the extended structure of (I). There are weak C—H⋯O inter­molecular inter­actions between one methyl group from the di­thiol­ate ligand and one of the carbonyl oxygen atoms, Table 1 ▸. These inter­actions result in the formation of dimers of (I), Fig. 3 ▸.

## Database survey   

Only one other crystal structure with 3,3-dimethyl-4-(propan-2-yl­idene)thietane-2,2-bis­(thiol­ate) is reported in the Cambridge Crystallographic Database (Groom & Allen, 2014 ▸). The compound is a mononuclear square-planar platinum(II) bis­(tri­phenyl­phosphine) complex (Okuma *et al.*, 2007 ▸).

A search of the Cambridge Crystallographic Database (Groom & Allen, 2014 ▸) returns eighteen hexa­carbonyldi-iron(I) complexes in which there is a bridging S—C—S di­thiol­ate moiety. The range of Fe—Fe distances for these compounds is 2.461 Å − 2.501 Å [average 2.482 Å] (Alvarez-Toledano *et al.*, 1999 ▸; Shi *et al.*, 2011 ▸). The Fe1—Fe2 distance in (I) of 2.4921 (4) Å falls within this range. The Fe—S distances for the database compounds range from 2.244 Å − 2.296 Å [average 2.271 Å] (Broadhurst *et al.*, 1982 ▸; Nekhaev *et al.*, 1991 ▸). All of the Fe—S distances in (I) [average 2.263 Å] fall within this range.

## Synthesis and crystallization   

A mixture of tetra­methyl-1,3-cyclo­butane­dithione (130 mg, 0.76 mmol) and Fe_3_(CO)_12_ (383 mg, 0.76 mmol) was dissolved in 15 ml dry toluene. The reaction mixture was refluxed for 2 h, and the solution color change from a green to a red was observed. After removal of the solvent under vacuum, the resulting residue was chromatographed by silica gel column eluting with hexa­ne–CH_2_Cl_2_ (10:1, *v*/*v*). The main red band was collected to get an orange–red solid (10 mg, 0.02 mmol, 3% yield). Crystals suitable for X-ray diffraction were grown by slow evaporation of hexane of the orange–red solid at room temperature.

## Refinement   

Crystal data, data collection and structure refinement details are summarized in Table 2 ▸. Methyl H atom positions were optimized by rotation about *R*—C bonds with idealized C—H, *R*⋯H and H⋯H distances and included as as riding idealized contributors [C—H_meth­yl_ = 0.98 Å with *U*_iso_(H) = 1.5*U*_eq_(C)]. The 001 reflection was omitted from the final refinement because it was obscured by the shadow of the beam stop.

## Supplementary Material

Crystal structure: contains datablock(s) I. DOI: 10.1107/S2056989015018496/wm5218sup1.cif

Structure factors: contains datablock(s) I. DOI: 10.1107/S2056989015018496/wm5218Isup2.hkl

Supporting information file. DOI: 10.1107/S2056989015018496/wm5218Isup3.cdx

CCDC reference: 1429290

Additional supporting information:  crystallographic information; 3D view; checkCIF report

## Figures and Tables

**Table 1:** Hydrogen-bond geometry (, )

*D*H*A*	*D*H	H*A*	*D* *A*	*D*H*A*
C13H13*B*O2^i^	0.98	2.56	3.334(2)	136

Symmetry code: (i) 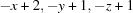.

**Table 2:** Experimental details

Crystal data	
Chemical formula	[Fe_2_(C_8_H_12_S_3_)(CO)_6_]
*M*_r_	484.12
Crystal system, space group	Triclinic, *P* 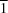
Temperature (K)	100
*a*, *b*, *c* ()	9.3619(10), 9.7681(11), 10.6249(12)
, , ()	88.092(6), 78.668(6), 76.559(6)
*V* (^3^)	926.51(18)
*Z*	2
Radiation type	Mo *K*
(mm^1^)	1.93
Crystal size (mm)	0.27 0.13 0.05

Data collection	
Diffractometer	Bruker Kappa APEXII CCD
Absorption correction	Integration (*SADABS*; Bruker, 2014 ▸)
*T*_min_, *T*_max_	0.752, 0.935
No. of measured, independent and observed [*I* > 2(*I*)] reflections	26313, 4095, 3603
*R*_int_	0.034
(sin /)_max_ (^1^)	0.643

Refinement	
*R*[*F* ^2^ > 2(*F* ^2^)], *wR*(*F* ^2^), *S*	0.022, 0.055, 1.04
No. of reflections	4095
No. of parameters	230
H-atom treatment	H-atom parameters constrained
_max_, _min_ (e ^3^)	0.46, 0.26

Computer programs: *APEX2*, *SAINT* and *XCIF* (Bruker, 2014 ▸), *SHELXTL* (Sheldrick, 2008 ▸), *SHELXT* (Sheldrick, 2015*a* ▸), *SHELXL2014* (Sheldrick, 2015*b* ▸), *CrystalMaker* (CrystalMaker, 1994 ▸) and *publCIF* (Westrip, 2010 ▸).

## supplementary crystallographic information

### Crystal data

**Table:**

[Fe_2_(C_8_H_12_S_3_)(CO)_6_]	*Z* = 2
*M**_r_* = 484.12	*F*(000) = 488
Triclinic, *P*1	*D*_x_ = 1.735 Mg m^−^^3^
*a* = 9.3619 (10) Å	Mo *K*α radiation, λ = 0.71073 Å
*b* = 9.7681 (11) Å	Cell parameters from 9920 reflections
*c* = 10.6249 (12) Å	θ = 2.3–27.1°
α = 88.092 (6)°	µ = 1.93 mm^−^^1^
β = 78.668 (6)°	*T* = 100 K
γ = 76.559 (6)°	Plate, orange
*V* = 926.51 (18) Å^3^	0.27 × 0.13 × 0.05 mm

### Data collection

**Table:**

Bruker Kappa APEXII CCD diffractometer	4095 independent reflections
Radiation source: fine-focus sealed tube	3603 reflections with *I* > 2σ(*I*)
Graphite monochromator	*R*_int_ = 0.034
profile data from φ and ω scans	θ_max_ = 27.2°, θ_min_ = 2.7°
Absorption correction: integration (*SADABS*; Bruker, 2014)	*h* = −12→11
*T*_min_ = 0.752, *T*_max_ = 0.935	*k* = −12→12
26313 measured reflections	*l* = −13→13

### Refinement

**Table:**

Refinement on *F*^2^	0 restraints
Least-squares matrix: full	Hydrogen site location: inferred from neighbouring sites
*R*[*F*^2^ > 2σ(*F*^2^)] = 0.022	H-atom parameters constrained
*wR*(*F*^2^) = 0.055	*w* = 1/[σ^2^(*F*_o_^2^) + (0.0274*P*)^2^ + 0.4094*P*] where *P* = (*F*_o_^2^ + 2*F*_c_^2^)/3
*S* = 1.04	(Δ/σ)_max_ = 0.001
4095 reflections	Δρ_max_ = 0.46 e Å^−^^3^
230 parameters	Δρ_min_ = −0.26 e Å^−^^3^

### Special details

**Table:**

Experimental. One distinct cell was identified using *APEX2* (Bruker, 2014). Twelve frame series were integrated and filtered for statistical outliers using *SAINT* (Bruker, 2014) then corrected for absorption by integration using *SAINT*/*SADABS* v2014/2 (Bruker, 2014) to sort, merge, and scale the combined data. No decay correction was applied.
Geometry. All e.s.d.'s (except the e.s.d. in the dihedral angle between two l.s. planes) are estimated using the full covariance matrix. The cell e.s.d.'s are taken into account individually in the estimation of e.s.d.'s in distances, angles and torsion angles; correlations between e.s.d.'s in cell parameters are only used when they are defined by crystal symmetry. An approximate (isotropic) treatment of cell e.s.d.'s is used for estimating e.s.d.'s involving l.s. planes.
Refinement. Structure was phased by direct methods (Sheldrick, 2015). Systematic conditions suggested the ambiguous space group. The space group choice was confirmed by successful convergence of the full-matrix least-squares refinement on *F*^2^. The final map had no significant features. A final analysis of variance between observed and calculated structure factors showed little dependence on amplitude and resolution.

### Fractional atomic coordinates and isotropic or equivalent isotropic displacement parameters (Å^2^)

**Table:**

	*x*	*y*	*z*	*U*_iso_*/*U*_eq_	
Fe1	0.76261 (3)	0.69641 (2)	0.81874 (2)	0.01268 (7)	
Fe2	0.54756 (3)	0.67026 (2)	0.72314 (2)	0.01297 (7)	
S1	0.78928 (4)	0.57564 (4)	0.63376 (4)	0.01365 (9)	
S2	0.66378 (4)	0.84946 (4)	0.67671 (4)	0.01170 (9)	
S3	0.98623 (4)	0.77978 (5)	0.51958 (4)	0.01510 (9)	
O1	1.06505 (15)	0.74224 (16)	0.81135 (13)	0.0298 (3)	
O2	0.79552 (19)	0.44486 (15)	0.97850 (14)	0.0394 (4)	
O3	0.60206 (15)	0.87309 (13)	1.04475 (12)	0.0228 (3)	
O4	0.49994 (16)	0.40404 (14)	0.84072 (15)	0.0342 (4)	
O5	0.30956 (14)	0.84235 (14)	0.91503 (13)	0.0236 (3)	
O6	0.37887 (15)	0.67444 (16)	0.51589 (13)	0.0289 (3)	
C1	0.9483 (2)	0.72664 (19)	0.81121 (16)	0.0194 (4)	
C2	0.7844 (2)	0.54209 (19)	0.91537 (17)	0.0229 (4)	
C3	0.66473 (19)	0.80602 (18)	0.95676 (17)	0.0158 (3)	
C4	0.5173 (2)	0.50717 (19)	0.79429 (18)	0.0217 (4)	
C5	0.39957 (19)	0.77459 (18)	0.83967 (17)	0.0176 (4)	
C6	0.4451 (2)	0.67316 (19)	0.59538 (17)	0.0193 (4)	
C7	0.80188 (18)	0.74138 (17)	0.55009 (16)	0.0126 (3)	
C8	0.78750 (18)	0.75972 (17)	0.40463 (15)	0.0133 (3)	
C9	0.94031 (18)	0.79590 (17)	0.36484 (16)	0.0145 (3)	
C10	1.01847 (19)	0.83374 (17)	0.25666 (16)	0.0152 (3)	
C11	0.7806 (2)	0.62209 (19)	0.34363 (17)	0.0189 (4)	
H11A	0.6862	0.5968	0.3818	0.028*	
H11B	0.8648	0.5469	0.3591	0.028*	
H11C	0.7865	0.6344	0.2510	0.028*	
C12	0.65819 (19)	0.8813 (2)	0.38437 (17)	0.0200 (4)	
H12A	0.6693	0.9681	0.4210	0.030*	
H12B	0.5630	0.8603	0.4269	0.030*	
H12C	0.6594	0.8933	0.2923	0.030*	
C13	0.9618 (2)	0.8528 (2)	0.13289 (17)	0.0205 (4)	
H13A	0.8732	0.8135	0.1409	0.031*	
H13B	1.0401	0.8039	0.0632	0.031*	
H13C	0.9352	0.9533	0.1138	0.031*	
C14	1.1685 (2)	0.8657 (2)	0.25251 (18)	0.0218 (4)	
H14A	1.1921	0.8566	0.3387	0.033*	
H14B	1.1662	0.9620	0.2225	0.033*	
H14C	1.2452	0.7994	0.1937	0.033*	

### Atomic displacement parameters (Å^2^)

**Table:**

	*U*^11^	*U*^22^	*U*^33^	*U*^12^	*U*^13^	*U*^23^
Fe1	0.01333 (12)	0.01381 (12)	0.01084 (12)	−0.00155 (9)	−0.00425 (9)	0.00130 (9)
Fe2	0.01163 (12)	0.01325 (12)	0.01452 (13)	−0.00397 (9)	−0.00246 (9)	0.00064 (9)
S1	0.0144 (2)	0.01203 (18)	0.0139 (2)	−0.00127 (15)	−0.00330 (15)	0.00014 (15)
S2	0.01227 (19)	0.01146 (18)	0.01142 (19)	−0.00226 (15)	−0.00305 (15)	0.00056 (15)
S3	0.01174 (19)	0.0236 (2)	0.01148 (19)	−0.00601 (16)	−0.00357 (15)	0.00040 (16)
O1	0.0193 (7)	0.0544 (9)	0.0193 (7)	−0.0119 (6)	−0.0086 (5)	0.0002 (6)
O2	0.0551 (10)	0.0243 (8)	0.0281 (8)	0.0052 (7)	−0.0018 (7)	0.0122 (6)
O3	0.0259 (7)	0.0243 (7)	0.0171 (7)	−0.0016 (6)	−0.0059 (5)	−0.0038 (5)
O4	0.0307 (8)	0.0213 (7)	0.0478 (9)	−0.0091 (6)	0.0012 (7)	0.0108 (7)
O5	0.0179 (6)	0.0262 (7)	0.0248 (7)	−0.0057 (5)	0.0016 (5)	−0.0053 (6)
O6	0.0243 (7)	0.0419 (8)	0.0265 (7)	−0.0134 (6)	−0.0117 (6)	−0.0014 (6)
C1	0.0213 (9)	0.0254 (9)	0.0107 (8)	−0.0020 (7)	−0.0054 (7)	0.0003 (7)
C2	0.0263 (10)	0.0218 (9)	0.0162 (9)	0.0022 (8)	−0.0030 (7)	−0.0004 (7)
C3	0.0159 (8)	0.0174 (8)	0.0166 (9)	−0.0047 (7)	−0.0087 (7)	0.0058 (7)
C4	0.0173 (9)	0.0211 (9)	0.0255 (10)	−0.0043 (7)	−0.0009 (7)	−0.0017 (8)
C5	0.0172 (9)	0.0181 (8)	0.0211 (9)	−0.0092 (7)	−0.0062 (7)	0.0034 (7)
C6	0.0165 (9)	0.0205 (9)	0.0208 (9)	−0.0067 (7)	0.0000 (7)	−0.0013 (7)
C7	0.0120 (8)	0.0135 (7)	0.0124 (8)	−0.0026 (6)	−0.0030 (6)	0.0009 (6)
C8	0.0127 (8)	0.0173 (8)	0.0096 (8)	−0.0018 (6)	−0.0030 (6)	−0.0007 (6)
C9	0.0146 (8)	0.0168 (8)	0.0129 (8)	−0.0029 (6)	−0.0047 (6)	−0.0008 (6)
C10	0.0154 (8)	0.0142 (8)	0.0149 (8)	−0.0013 (6)	−0.0026 (6)	−0.0016 (6)
C11	0.0202 (9)	0.0245 (9)	0.0145 (8)	−0.0093 (7)	−0.0043 (7)	−0.0025 (7)
C12	0.0166 (9)	0.0281 (10)	0.0135 (8)	0.0000 (7)	−0.0053 (7)	0.0044 (7)
C13	0.0194 (9)	0.0275 (10)	0.0134 (8)	−0.0036 (7)	−0.0028 (7)	0.0033 (7)
C14	0.0185 (9)	0.0269 (10)	0.0213 (9)	−0.0091 (7)	−0.0021 (7)	0.0010 (8)

### Geometric parameters (Å, º)

**Table:**

Fe1—C2	1.7900 (18)	O6—C6	1.140 (2)
Fe1—C3	1.8047 (18)	C7—C8	1.578 (2)
Fe1—C1	1.8158 (19)	C8—C9	1.529 (2)
Fe1—S2	2.2636 (5)	C8—C12	1.529 (2)
Fe1—S1	2.2675 (5)	C8—C11	1.532 (2)
Fe1—Fe2	2.4921 (4)	C9—C10	1.327 (2)
Fe2—C4	1.7986 (19)	C10—C14	1.500 (2)
Fe2—C5	1.8013 (19)	C10—C13	1.502 (2)
Fe2—C6	1.8054 (19)	C11—H11A	0.9800
Fe2—S2	2.2601 (5)	C11—H11B	0.9800
Fe2—S1	2.2624 (5)	C11—H11C	0.9800
S1—C7	1.8376 (17)	C12—H12A	0.9800
S2—C7	1.8365 (17)	C12—H12B	0.9800
S3—C9	1.7725 (17)	C12—H12C	0.9800
S3—C7	1.8159 (17)	C13—H13A	0.9800
O1—C1	1.139 (2)	C13—H13B	0.9800
O2—C2	1.141 (2)	C13—H13C	0.9800
O3—C3	1.138 (2)	C14—H14A	0.9800
O4—C4	1.139 (2)	C14—H14B	0.9800
O5—C5	1.140 (2)	C14—H14C	0.9800
			
C2—Fe1—C3	91.53 (8)	C8—C7—S3	92.82 (10)
C2—Fe1—C1	97.19 (9)	C8—C7—S2	120.98 (11)
C3—Fe1—C1	98.73 (8)	S3—C7—S2	115.14 (9)
C2—Fe1—S2	156.52 (7)	C8—C7—S1	121.18 (11)
C3—Fe1—S2	94.05 (5)	S3—C7—S1	115.21 (8)
C1—Fe1—S2	104.45 (6)	S2—C7—S1	93.42 (8)
C2—Fe1—S1	94.27 (6)	C9—C8—C12	112.78 (14)
C3—Fe1—S1	156.84 (5)	C9—C8—C11	112.89 (14)
C1—Fe1—S1	102.74 (6)	C12—C8—C11	111.99 (14)
S2—Fe1—S1	72.347 (18)	C9—C8—C7	93.06 (12)
C2—Fe1—Fe2	100.05 (7)	C12—C8—C7	112.71 (13)
C3—Fe1—Fe2	100.38 (5)	C11—C8—C7	112.13 (13)
C1—Fe1—Fe2	153.78 (5)	C10—C9—C8	135.38 (15)
S2—Fe1—Fe2	56.505 (14)	C10—C9—S3	128.31 (14)
S1—Fe1—Fe2	56.527 (14)	C8—C9—S3	96.26 (11)
C4—Fe2—C5	92.90 (8)	C9—C10—C14	121.21 (16)
C4—Fe2—C6	97.79 (8)	C9—C10—C13	123.00 (16)
C5—Fe2—C6	98.27 (8)	C14—C10—C13	115.75 (15)
C4—Fe2—S2	156.52 (6)	C8—C11—H11A	109.5
C5—Fe2—S2	92.61 (6)	C8—C11—H11B	109.5
C6—Fe2—S2	103.96 (6)	H11A—C11—H11B	109.5
C4—Fe2—S1	93.49 (6)	C8—C11—H11C	109.5
C5—Fe2—S1	154.57 (6)	H11A—C11—H11C	109.5
C6—Fe2—S1	105.19 (6)	H11B—C11—H11C	109.5
S2—Fe2—S1	72.505 (18)	C8—C12—H12A	109.5
C4—Fe2—Fe1	99.98 (6)	C8—C12—H12B	109.5
C5—Fe2—Fe1	97.92 (6)	H12A—C12—H12B	109.5
C6—Fe2—Fe1	155.23 (6)	C8—C12—H12C	109.5
S2—Fe2—Fe1	56.639 (13)	H12A—C12—H12C	109.5
S1—Fe2—Fe1	56.720 (15)	H12B—C12—H12C	109.5
C7—S1—Fe2	90.00 (5)	C10—C13—H13A	109.5
C7—S1—Fe1	86.81 (5)	C10—C13—H13B	109.5
Fe2—S1—Fe1	66.753 (15)	H13A—C13—H13B	109.5
C7—S2—Fe2	90.10 (5)	C10—C13—H13C	109.5
C7—S2—Fe1	86.96 (5)	H13A—C13—H13C	109.5
Fe2—S2—Fe1	66.856 (15)	H13B—C13—H13C	109.5
C9—S3—C7	77.86 (8)	C10—C14—H14A	109.5
O1—C1—Fe1	176.96 (16)	C10—C14—H14B	109.5
O2—C2—Fe1	178.63 (18)	H14A—C14—H14B	109.5
O3—C3—Fe1	178.75 (16)	C10—C14—H14C	109.5
O4—C4—Fe2	178.76 (18)	H14A—C14—H14C	109.5
O5—C5—Fe2	177.61 (15)	H14B—C14—H14C	109.5
O6—C6—Fe2	179.05 (16)		
			
C9—S3—C7—C8	0.66 (9)	S3—C7—C8—C12	−117.02 (13)
C9—S3—C7—S2	−125.68 (10)	S2—C7—C8—C12	4.7 (2)
C9—S3—C7—S1	127.24 (10)	S1—C7—C8—C12	121.10 (14)
Fe2—S2—C7—C8	99.53 (12)	S3—C7—C8—C11	115.51 (12)
Fe1—S2—C7—C8	166.35 (13)	S2—C7—C8—C11	−122.78 (14)
Fe2—S2—C7—S3	−150.28 (8)	S1—C7—C8—C11	−6.38 (19)
Fe1—S2—C7—S3	−83.46 (8)	C12—C8—C9—C10	−60.3 (3)
Fe2—S2—C7—S1	−30.32 (6)	C11—C8—C9—C10	67.9 (2)
Fe1—S2—C7—S1	36.49 (5)	C7—C8—C9—C10	−176.5 (2)
Fe2—S1—C7—C8	−99.43 (12)	C12—C8—C9—S3	116.98 (13)
Fe1—S1—C7—C8	−166.14 (12)	C11—C8—C9—S3	−114.84 (13)
Fe2—S1—C7—S3	150.19 (8)	C7—C8—C9—S3	0.77 (11)
Fe1—S1—C7—S3	83.47 (8)	C7—S3—C9—C10	176.85 (18)
Fe2—S1—C7—S2	30.29 (6)	C7—S3—C9—C8	−0.68 (10)
Fe1—S1—C7—S2	−36.43 (5)	C8—C9—C10—C14	178.40 (17)
S3—C7—C8—C9	−0.74 (11)	S3—C9—C10—C14	1.9 (3)
S2—C7—C8—C9	120.97 (13)	C8—C9—C10—C13	0.8 (3)
S1—C7—C8—C9	−122.63 (13)	S3—C9—C10—C13	−175.74 (13)

### Hydrogen-bond geometry (Å, º)

**Table:**

*D*—H···*A*	*D*—H	H···*A*	*D*···*A*	*D*—H···*A*
C13—H13*B*···O2^i^	0.98	2.56	3.334 (2)	136

Symmetry code: (i) −*x*+2, −*y*+1, −*z*+1.
